# Acute Encephalitis During a SARS-CoV-2 Infection: A Case Report

**DOI:** 10.7759/cureus.62009

**Published:** 2024-06-09

**Authors:** Yousfi Samah, Ansam Milhi, Sanae Elhasnaoui, Yassine Mebrouk

**Affiliations:** 1 Department of Neurology, Mohammed VI International University Hospital, Oujda, MAR; 2 Faculty of Medicine, Mohammed First University, Oujda, MAR; 3 Department of Neurology, Centre Hospitalo-Universitaire Mohammed VI, Oujda, MAR

**Keywords:** immune-mediated, cytokines, neurological tropism, encephalitis, neurological complications, covid-19

## Abstract

It was in December 2019 that the coronavirus causing COVID-19 was first detected in Wuhan, China. Although the primary clinical presentation is respiratory disease, an increasing number of reports worldwide have noted various neurological manifestations, such as acute encephalitis.

We present a case of a 49-year-old female admitted with afebrile impaired consciousness, diagnosed with acute encephalitis and a severe infection of COVID-19. Clinical and radiological improvement was observed following treatment with corticosteroids.

## Introduction

The outbreak of COVID-19 triggered a global public health emergency [1]. As of August 26th, 2021, there are a total of 214 million confirmed cases and 4.46 million confirmed deaths worldwide. The COVID-19 pandemic has caused unprecedented hazards to public health and the global economy, among others [2]. The neurological manifestations associated with COVID-19 are notably intricate, characterized by a spectrum of clinical, biological, and radiological presentations linked to inflammatory cerebral involvement induced by this virus, a phenomenon that is infrequently documented. Moreover, a favorable response to immunotherapy may suggest the presence of a dysimmune para-infectious mechanism [3].

SARS-CoV-2 can disseminate beyond its initial site of infection. This emerging infectious illness has been linked to a range of health complications, including acute respiratory distress syndrome (ARDS), severe metabolic disorders, thromboembolic events, severe acute tubular necrosis, electrolyte imbalances, neurological disorders, and cardiac events such as myocarditis and arrhythmias. COVID-19 demonstrates neuroinvasive properties, highlighting the CNS as a significant target [3].

Various neurological symptoms and complications have been reported in conjunction with COVID-19, including but not limited to anosmia, ageusia, encephalitis, and Guillain-Barré syndrome. However, many others remain a mystery.

Despite a growing number of reported cases, encephalitis related to COVID-19 infection has not been adequately characterized. Many studies have speculated that SARS-CoV-2 may cause neurological damage. With this case report, we provide clinical evidence showing the CNS involvement of SARS-CoV-2 [4].

## Case presentation

In this report, we present the case of a 49-year-old female patient who presented to the emergency room with severe afebrile consciousness disorders, following an episode in the past two weeks of fever, fatigue, severe headache, nausea, a dry cough, and worsening lethargy, with a PCR test for SARS-CoV-2 RNA on a nasopharyngeal swab that was initially negative.

Past medical history shows five years of type 2 diabetes treated with oral hypoglycemic medications and a vaccination against COVID-19 by Sinopharm vaccine: the first dose on May 21st and the second on June 19th. Surgical, drug, and family history were unremarkable.

On the initial physical examination, the patient was afebrile and unconscious with a Glasgow Coma Scale (GCS) of 3/15, did not follow commands or respond to painful stimuli, and had pupils that were equal and reactive. Vital signs showed elevated blood pressure (179/92 mmHg), oxygen saturation at 70% in ambient air and 85% under a high concentration mask with 15 L/min, heart rate at 85/min, and respiratory frequency at 28 cycles/min.

The neurological exam showed hypotonia of all four limbs, decreased deep tendon reflexes, normal plantar reflex, and no signs of meningeal irritation. The extraneurological exam found sonorous rhonchi.

The patient’s initial laboratory blood testing revealed an elevated white blood count at 15,520/mm^3^ with polys at 13,370/mm^3^, lymphopenia at 700/mm^3^, elevated C-reactive protein at 62.7 mg/L, procalcitonin at 0.48 ng/mL, blood glucose at 3.24 g/L with negative acetone, high D-dimers at 6.24 ng/mL, ferritin within normal limits at 111 ng/mL, hypernatremia at 150 mmol/L, and blood gas analysis showing pH at 7.44, pCO2 at 68, pO2 at 68, SAO2 at 94%, HCO3 at 29.4, and lactate at 1.84 (Table 1).

**Table 1:** Results of the patient's biological assessments.

Laboratory Parameter Names	Observed Values	Reference Range
Blood Count		
Haemoglobin	14 g/dL	Men: 13-18 g/dL, Women: 12-16 g/dL
Mean Corpuscular Volume (MCV)	91 fL	80-95 fL
Mean Corpuscular Hemoglobin Concentration (MCHC)	31%	30-35%
White Blood Cell	15,520/mm³	4,000-10,000 /mm³
Platelets	290 g/L	150-350 g/L
Lymphocytes	700/mm³	1,500-4,000 /mm³
Neutrophils	13,370/mm³	1,500-7,500/mm³
Eosinophils	60/mm³	50-500/mm³
Monocytes	240/mm³	200-800/mm³
CRP Analysis	62.7 mg/L	<4 mg/L
Procalcitonin	0.48 ng/mL	<0.5 ng/mL
Blood Glucose	3.24 g/L	0.70-1.10 g/L
Acetone	1 mg/dL	
D-dimers	6.24 µg/mL	<0.5 µg/mL
Ferritin	111 ng/mL	7-132 ng/mL
Blood Electrolytes		
Natremia	150 mEq/L	135-145 mEq/L
Blood Gas		
pH	7.44	7.38-7.42
PCO2	68 mm Hg	<40 mm Hg
PO2	68 mm Hg	>75 mm Hg
SAO2	94%	95-100%
HCO3	29.4 mmol/L	22-28 mmol/L
Lactate	1.84 mmol/L	0.5-1.5 mmol/L

**Figure 1:**
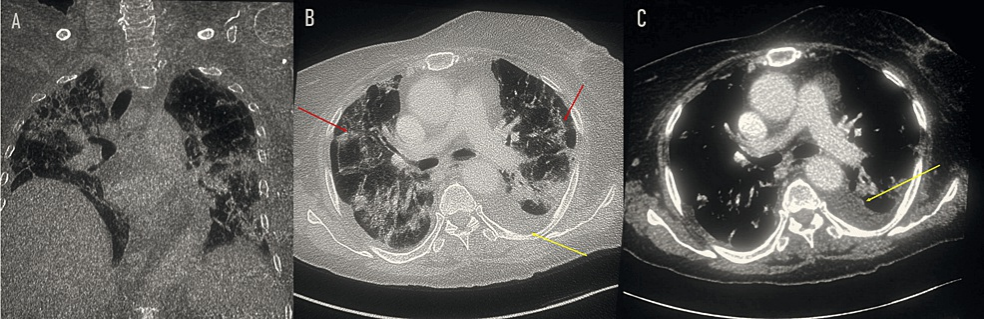
Thoracic scan: coronal section (A), axial section with non-enhanced sequence (B), and axial section with enhanced sequence (C) showing alveolar interstitial syndrome with fairly severe involvement (red arrow) and a left pleural effusion blade (yellow arrow). No scan signs related to pulmonary embolism. CORADS: COVID-19 Reporting and Data System.

Meanwhile, a brain CT scan (non-contrast) revealed hypodense areas in the thalamic and external capsule bilaterally. A chest CT (with and without contrast) showed the presence of bilateral alveoli and interstitial pneumopathy consistent with COVID-19 pneumonia (CORADS 4) (Figure 1).

**Figure 2:**
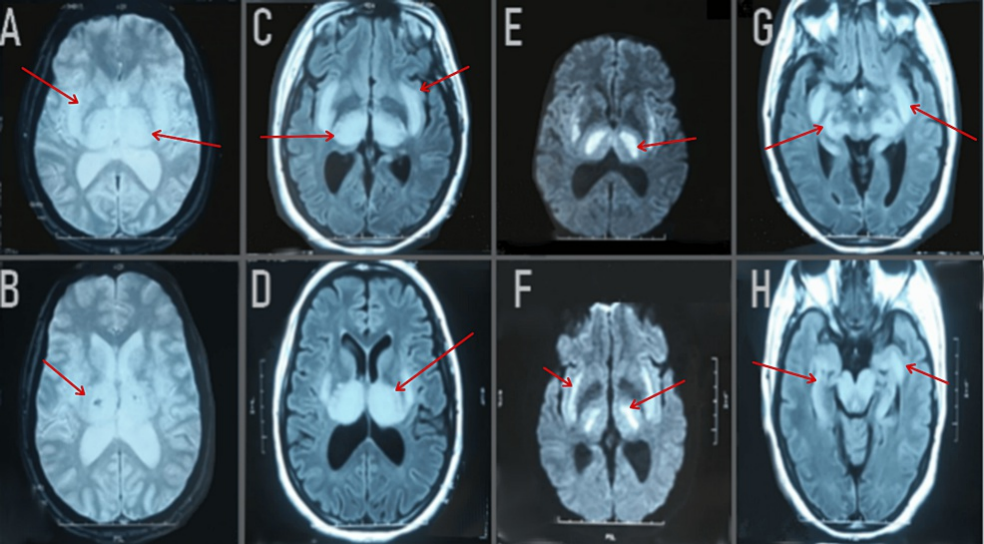
Axial FLAIR images (C, D, G, H) showed bilateral hypersignal abnormalities (red arrow) in the thalamus, posterior limb of the internal capsule, external capsule, putamen, hypothalamus, pons, and bulb. Axial images with gadolinium demonstrated no abnormal contrast enhancement (not shown). T2*-weighted images (A, B) showed bilateral thalamic hemorrhagic lesions. Diffusion-weighted imaging (E, F) showed a hypersignal B1000 with an apparent diffusion coefficient decrease. FLAIR: Fluid-Attenuated Inversion Recovery; T2: T2-weighted imaging; DWI: Diffusion Weighted Imaging; ADC: Apparent Diffusion Coefficient.

On the same day of the patient’s admission, a brain MRI was carried out; the results revealed the presence of a T2-fluid attenuated inversion recovery (FLAIR) weighted hypersignal in the bithalamic-peduncle hippocampus and right internal temporal lobe. These lesions are hypointense on T1, showed no contrast enhancement, and diffusion-weighted imaging displayed a hypersignal B1000 with an apparent diffusion coefficient decrease (Figure 2).

The patient was later admitted to the ICU, where she was intubated, sedated, and ventilated with FiO2 at 100% and SaO2 at 94%. She received five days of bolus steroid therapy as well as COVID-19 protocol treatment.

Seven days after starting steroid therapy, the patient was extubated, underwent a tracheostomy, and with a SaO2 of 94% in ambient air, was transferred to the neurology department for further care. Once admitted to the neurology department, the patient’s GCS improved to 9/15, while the rest of her neurological examination remained the same.

**Figure 3:**
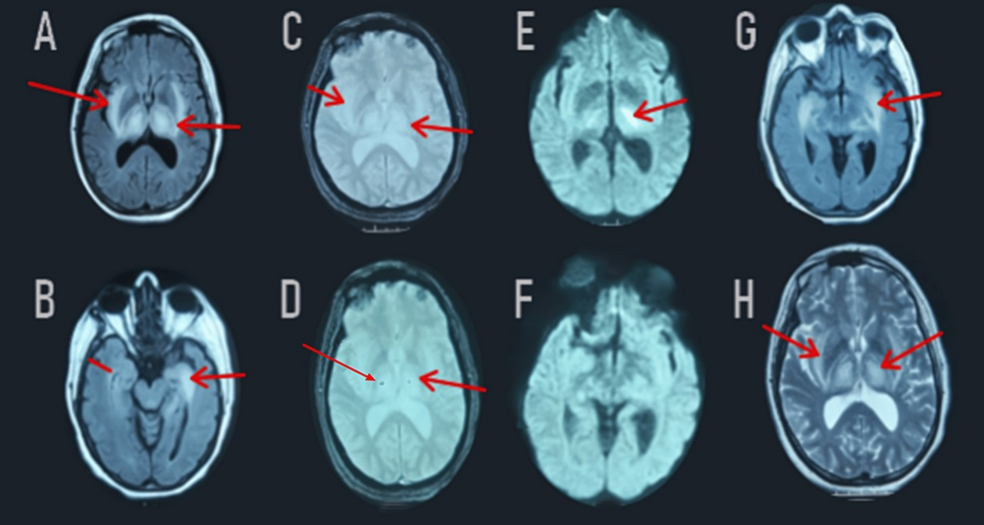
Follow-up MRI revealed significant regression of the FLAIR-weighted hypersignal in the two thalami, the cerebral peduncles, the bulb, the hippocampus, and the right internal temporal areas (A, B, G). Diffusion-weighted imaging (E, F) showed a decrease in the apparent diffusion coefficient on the periphery (red arrow). Post-gadolinium images demonstrated contrast enhancement in the two thalami (not shown). T2*-weighted images (C, D) showed a stable appearance of the bilateral thalamic hemorrhagic lesions (red arrow). FLAIR: Fluid-Attenuated Inversion Recovery; T2: T2-weighted imaging; DWI: Diffusion Weighted Imaging; ADC: Apparent Diffusion Coefficient.

Shortly thereafter, another brain MRI was performed, revealing a regression in the bithalamic-peduncle hippocampus and right internal temporal lesions. The discovery of bithalamic contrast lesions was explained by a rupture of the blood-brain barrier (Figure 3).

**Figure 4:**
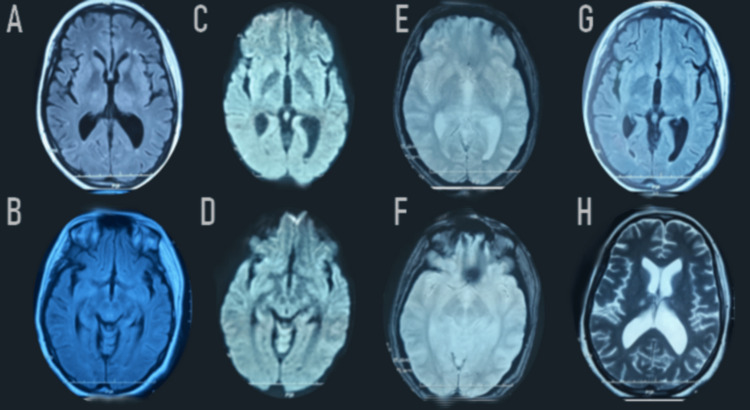
Remarkable regression of the FLAIR-weighted (A-B-G) and diffusion-weighted (C-D) bilateral hypersignal in the thalamus. Extinction of the bilateral signal abnormalities in the external capsule, posterior limb of the internal capsule, and in the pons. Axial T2*-weighted images (E-F) showed bilateral thalamic hemorrhagic lesions and in the posterior part of the putamen, as well as the presence of minimal intraventricular subarachnoid hemorrhage in the falx cerebri and cerebellar tentorium. FLAIR: Fluid-Attenuated Inversion Recovery; T2: T2-weighted imaging; DWI: Diffusion Weighted Imaging.

The patient continued to receive nursing care procedures and strict surveillance of her vitals within the neurology department, and her status remained stable. A second surveillance brain MRI showed a satisfactory evolution of the brain lesions, revealing residual lesions on the thalamic nuclei and bilateral pallidum, and also the presence of minimal intraventricular subarachnoid hemorrhage (Figure 4).

The patient’s status remains stable with no further progression in the neurology department of the University Hospital Center of Oujda.

## Discussion

With the advent and increase in cases of SARS-CoV-2 infection, numerous systemic complications have been described, including involvement of the CNS and peripheral nervous systems (PNS). Many studies have indicated that coronaviruses possess neurotropic and neuroinvasive properties, even in the absence of pulmonary symptoms. Cerebrovascular disease, encephalitis, myelitis, and cases of anosmia and ageusia represent the most common neurological manifestations related to SARS-CoV-2. Other nonspecific neurological signs such as headaches, dizziness, myalgia, neuropathic pain, and isolated epileptic seizures have been linked to this infection in some patients [5].

Several neurological clinical manifestations have been observed in patients with COVID-19 [6]. In a systematic review and meta-analysis with a pooled sample size of 13,480 patients, neurological manifestations were frequent, with around 20% of patients reporting myalgia, taste impairment, or smell impairment; and about 10% complaining of headache, dizziness, or encephalopathy [7].

Neurological manifestations have become increasingly apparent as the COVID-19 pandemic continues, and sporadic cases of COVID-related encephalitis have been reported. We describe a case of autoimmune encephalitis during a severe COVID-19 infection presented with impaired consciousness.

Encephalitis is defined as inflammation of the brain parenchyma associated with neurological dysfunction. Ellul MA et al. propose criteria to determine the association with COVID-19: a confirmed association is identified when the SARS-CoV-2 PCR test is positive in the cerebrospinal fluid, while a probable association is established when the SARS-CoV-2 PCR test is positive in a sample taken from outside the nervous system, and there is no other apparent cause. The symptomatology predominantly involves a disturbance of consciousness in 78% of cases, which could lead to the requirement for intensive care [8].

The abnormalities and neurological damage observed in COVID-19 can be explained by two phenomena. The first mechanism involves hematogenous dissemination through lesions in the blood-brain barrier or direct invasion via the ethmoid bone, near the olfactory bulb. The second mechanism is associated with the cytokine storm [9]. The entry of SARS-CoV-2 into human cells is commonly thought to be mediated by the interaction of the spike protein with the angiotensin-converting enzyme 2 (ACE2) receptor present in human airway epithelia, vascular endothelia, retina, smooth muscle, and also in the brain, particularly in the brainstem (both in neurons and glia), the cerebrum, and cerebellum. Several factors may be associated with COVID-related encephalopathies, including severe hypoxemia, inflammation (cytokine storm), acute cerebrovascular disease, direct viral invasion, and non-convulsive status epilepticus [10].

Various risk factors for encephalitis as a complication of COVID-19 were clarified. Certain demographic factors potentially heighten the risk of complications associated with COVID-19 infection, including the development of encephalitis, notably advanced age and associated comorbidities [11].

Patients with encephalitis and encephalopathy often have non-specific imaging patterns including leptomeningeal enhancement [10], acute hemorrhagic necrotizing encephalopathy, diffuse leukoencephalopathy [12], and increased white matter signal intensities after accounting for age, acute disseminated encephalomyelitis (ADEM), and posterior reversible encephalopathy syndrome (PRES) [13].

Common MRI brain findings in acute hemorrhagic necrotizing encephalopathy consist of multiple T2-hyperintense symmetrical lesions most frequently found in the thalami as well as in the putamina, periventricular white matter, cerebellum, and brainstem tegmentum. These findings are similar to our case. Hemorrhage and cavitation may also be present. Contrast material-enhanced images may demonstrate a ring of contrast enhancement [14]. The extent of lesions on MRI, tissue loss, involvement of the brainstem, and the presence of hemorrhage represent factors indicating a poor prognosis [15]. However, the normality of brain imaging in cases of encephalitis complicating COVID-19 is likely due to the early timing of imaging or to milder encephalitis [16].

In this case report, our 49-year-old patient developed neurological autoimmune complications that presented clinically as severe afebrile consciousness disorders, with severe pulmonary lesions revealed on the chest CT and biological anomalies, and appeared on the brain MRI as bithalamic-peduncle hippocampus and right internal temporal lesions, giving a very particular image. Our case emphasizes the importance of considering encephalitis due to COVID-19 infection in the differential diagnosis for patients presenting with similar neurological symptoms and MRI findings, in order to avoid wrongful or delayed diagnosis and ensure rapid treatment.

The evolution of this case was characterized by the improvement of the patient's neurological status (GCS) and significant regression of the brain lesions on MRI after the patient received 5 days of steroid bolus therapy and protocol treatment for COVID-19. However, the patient's status remains stationary with no further improvements noted.

## Conclusions

This clinical case argues for a possible relationship between SARS-CoV-2 infection and autoimmune encephalitis. The importance of interdisciplinary collaboration among neurologists, infectious disease specialists, and immunologists in managing complex cases of autoimmune encephalitis related to COVID-19 is crucial.

Neurological manifestations during SARS-CoV-2 infection are common and frequently severe. The significant heterogeneity in clinical, radiological, and pathological findings suggests that various pathophysiological mechanisms are involved. The most common manifestations, encephalopathies and cerebral infarctions, are at least partly linked to sepsis and hypoxia.

Faced with any severe neurological symptoms, a brain MRI should be performed to search for cerebral abnormalities that could potentially benefit from a steroid bolus treatment.

Nonetheless, the lack of a typical CSF profile for viral encephalitis, along with negative PCR results for SARS-CoV-2 in the CSF, complicates the diagnosis of SARS-CoV-2-associated encephalitis.
